# Circulating exosomal mir-16-2-3p is associated with coronary microvascular dysfunction in diabetes through regulating the fatty acid degradation of endothelial cells

**DOI:** 10.1186/s12933-024-02142-0

**Published:** 2024-02-09

**Authors:** Yihai Liu, Chongxia Zhong, Shan Chen, Yanan Xue, Zhonghai Wei, Li Dong, Lina Kang

**Affiliations:** 1 Department of Cardiology, Affiliated Drum Tower Hospital of Nanjing University Medical School, Nanjing, 210009 China; 2 Department of General Medicine, Affiliated Drum Tower Hospital of Nanjing University Medical School, Nanjing, 210009 China; 3 Department of Geriatrics, Nanjing Central Hospital, Nanjing, 210018 China

**Keywords:** miR-16-2-3p, Diabetes, Coronary microvascular dysfunction, Exosomes, Microvascular endothelial cells

## Abstract

**Background:**

Coronary microvascular dysfunction (CMD) is a frequent complication of diabetes mellitus (DM) characterized by challenges in both diagnosis and intervention. Circulating levels of microRNAs are increasingly recognized as potential biomarkers for cardiovascular diseases.

**Methods:**

Serum exosomes from patients with DM, DM with coronary microvascular dysfunction (DM-CMD) or DM with coronary artery disease (DM-CAD) were extracted for miRNA sequencing. The expression of miR-16-2-3p was assessed in high glucose-treated human aortic endothelial cells and human cardiac microvascular endothelial cells. Fluorescence in situ hybridization (FISH) was used to detect miR-16-2-3p within the myocardium of db/db mice. Intramyocardial injection of lentivirus overexpressing miR-16-2-3p was used to explore the function of the resulting gene in vivo. Bioinformatic analysis and in vitro assays were carried out to explore the downstream function and mechanism of miR-16-2-3p. Wound healing and tube formation assays were used to explore the effect of miR-16-2-3p on endothelial cell function.

**Results:**

miR-16-2-3p was upregulated in circulating exosomes from DM-CMD, high glucose-treated human cardiac microvascular endothelial cells and the hearts of db/db mice. Cardiac miR-16-2-3p overexpression improved cardiac systolic and diastolic function and coronary microvascular reperfusion. In vitro experiments revealed that miR-16-2-3p could regulate fatty acid degradation in endothelial cells, and ACADM was identified as a potential downstream target. MiR-16-2-3p increased cell migration and tube formation in microvascular endothelial cells.

**Conclusions:**

Our findings suggest that circulating miR-16-2-3p may serve as a biomarker for individuals with DM-CMD. Additionally, miR-16-2-3p appears to alleviate coronary microvascular dysfunction in diabetes by modulating ACADM-mediated fatty acid degradation in endothelial cells.

**Supplementary Information:**

The online version contains supplementary material available at 10.1186/s12933-024-02142-0.

## Introduction

Diabetes mellitus (DM) has emerged as a global health concern with a higher prevalence in recent years [1]. Despite being a disease with diverse vascular complications influencing the heart, nerve, kidney and retina, these complications are receiving more attention than the disease itself [2–4]. These vascular complications are categorized as macrovascular disease, which causes damage to arteries leading to stroke, coronary heart disease and necrosis; and microvascular disease, which damages small vessels, resulting in retinopathy, nephropathy, neuropathy and coronary microvascular dysfunction (CMD) [5].

CMD involves structural and functional alterations in coronary microcirculation, causing impaired coronary blood flow and ultimately resulting in myocardial ischemia [6]. It is highly prevalent in type 2 diabetic patients without overt cardiovascular disease. CMD was strongly associated with an increased risk of all-cause mortality and MACE in diabetic patients [7]. Currently, assessing CMD involves invasive techniques such as coronary angiography and noninvasive techniques such as transthoracic echocardiography, positron emission tomography, and cardiac magnetic resonance. However, the former is limited by high cost and complexity, while the latter lacks accuracy [8]. Therefore, identifying diagnostic markers for CMD in diabetic patients and determining the underlying pathophysiological mechanism are crucial.

Exosomes are secreted by various tissue cells and play a role in mediating intercellular communication through their contents, including nucleic acids, proteins and lipids [9, 10]. MicroRNAs (miRNAs) are short noncoding RNAs capable of regulating a wide array of biological processes [11]. A previous study confirmed the diagnostic potential of circulating exosomal miRNAs in cardiovascular diseases [12]. Given the systemic nature of diabetic complications, we hypothesized that circulating exosomal miRNAs could serve as key diagnostic, prognostic and personalized treatments for CMD in diabetic patients.

In this study, we isolated exosomes from serum and profiled the enrichment of miRNAs by sequencing. We identified miR-16-2-3p as a novel biomarker in DM-CMD and investigated its expression, function and downstream targets in silico and via in vitro and in vivo analyses.

## Materials and methods

### Study population

Patients were enrolled from the Cardiovascular Department of Nanjing Drum Hospital between May 2021 and May 2022. The study protocol was approved by the Ethics Committee of Nanjing Drum Hospital (2023-416-02) and adhered to the Declaration of Helsinki. The participants were divided into 3 groups according to the inclusion/exclusion criteria: the DM group, the DM-CMD group and the DM-CAD group (*n* = 3 each group). The diagnosis of diabetes followed the 1999 WHO diagnostic criteria [13] and included the following typical diabetic symptoms: (1) fasting blood glucose ≥ 7.0 mmol/L, (2) HbA1c ≥ 6.5%, and (3) random blood glucose ≥ 11.1 mmol/L. The diagnosis of CMD included the presence of labor-induced chest pain or tightness and objective evidence of myocardial ischemia (meeting at least one of the following criteria): (a) dynamic changes in ST-T during symptom onset; (b) positive results on the treadmill exercise test; (c) reversible ischemic changes observed in myocardial nuclide perfusion imaging during exercise/drug loading; (d) no obstruction in the coronary arteries from angiography. CAD diagnosis was established through coronary angiography or coronary CTA, which indicated stenosis in the lumen of the main coronary artery or branches with a diameter greater than 2 mm but less than 50%. All patients were excluded if they had (1) heart failure (ejection fraction, EF < 50%), (2) renal failure (estimated glomerular filtration rate, eGFR < 60 ml/min*1*73 m2), or (3) malignant tumors. Clinical characteristics, including age, sex, BMI, smoking status, hypertension status and the use of antidiabetic drugs, were collected. Peripheral blood samples were collected to analyze fasting glucose levels, HbA1c levels, eGFRs, total cholesterol levels, and triglyceride levels.

### Exosome isolation and characterization

Serum exosomes were isolated using ultracentrifugation as previously described with modifications [14]. Briefly, the serum was obtained by centrifugation at 3000 × g for 15 min to remove the cell debris and platelets. Microvesicles were pelleted at 10,000 × g for 30 min, after which the exosomes were further purified from the supernatant by ultracentrifugation at 100,000 × g for 60 min. Following isolation, the exosomes were diluted in 100 µL of filtered PBS and stored at − 80 °C.

Exosomes were dissolved in lysis buffer and quantified using a BCA analysis kit (Thermo Fisher Scientific, USA). Western blotting was used to detect exosomal markers, including TSG101, HSP70, CD63 and CD9. Exosomes were fixed in 2.5% glutaraldehyde at 4 °C, dehydrated with gradient alcohol and embedded in epoxy resin. Sections were stained with uranyl acetate and citrate acid lead. The images were captured under a transmission electron microscope (JEM-1010, Japan). For nanoparticle tracking analysis, samples were loaded into the sample chamber of an NS500 unit (NanoSight, UK), and five 1-min videos of each sample were recorded. The data analysis was performed with NTA 2.3 software, and the size and concentration of the particles were calculated.

### miRNA sequencing

The raw reads from small RNA sequencing were generated in fastq format, and the linker sequence was removed using Cutadapt. Fastx_toolkit (version 0.0.13) software was used to perform Q20 quality control on the sequences, and sequences with a Q20 of 80 or above were retained. The NGSQC toolkit (version 2.3.2) was subsequently used to filter out reads containing N-bases. The resulting high-quality clean reads were used for subsequent analysis. Statistics were performed on the length distribution of the clean reads to initially assess the distribution of small RNAs in the sample. Using blastn software, the clean reads were compared with those in the Rfam (version 10.0) database to annotate sequences such as rRNA, snRNA, snoRNA, and tRNA. Using RepeatMasker software, the filtered sequences were compared to those in the repeat database to identify possible repeats. Small RNA sequences on unannotated devices were predicted using miRDeep2 software for new miRNAs, and secondary structures of miRNAs were predicted using RNAfold software. The miRNA expression calculation used TPM (transcript per million) to calculate the metric, where TPM = number of reads per miRNA/total sample alignment read number ×106. The miRNA array data have been deposited in the Gene Expression Omnibus under accession number GSE234464.

### Bioinformatics analysis

For each group, the miRNAs with a mean count greater than 2 in at least one group were screened for subsequent differential expression analysis. Using the DESeq2 algorithm in the R package, miRNAs with a *P* value < 0.05 and a fold change > 2 were screened. The differences between DM-CMD and DM were compared to screen out the upregulated miRNAs in DM-CMD, and the differences between DM-CAD and DM were compared to screen out the upregulated miRNAs in DM-CAD. Enrichment analysis was performed to explore the molecular function and underlying targets of the differentially expressed miRNAs using miRPath.

### Animals

Male db/db mice aged 6–8 weeks were purchased from the Nanjing Model Animal Research Institute (Nanjing, China). The animals were adaptively raised in the animal room for 1 week at 23 °C with 55–60% humidity and a 12-hour light/dark cycle. We used 3 additional bks of mice to observe the difference in miR-16-2-3p expression as a control. Mice had access to drinking water and food ad libitum. Then, the db/db mice were randomly divided into 2 groups (*n* = 5 per group). The Micro group received a myocardial injection of lentivirus overexpressing miR-16-2-3p, and the Sham group received an empty vector. One month later, blood glucose levels, cardiac function, and microvascular perfusion were determined. The animal experiments were approved by the Animal Ethics Committee of Nanjing Drum Hospital, and all procedures conformed to the guidelines from Directive 2010/63/EU of the European Parliament on the protection of animals used for scientific purposes or the NIH Guide for the Care and Use of Laboratory Animals.

The miR-16-2-3p sequence was subcloned and inserted into an IRES-containing GFP-expressing plasmid, which was subsequently packaged in lentiviral vectors at a concentration of 5 × 10^8^ TU/ml. The intramyocardial injections were performed via a 30-gauge needle using a 20 µl micromanipulator (Hamilton, USA). A volume of 20 µl was injected into three distinct areas in the anterior wall of the left ventricle.

### Echocardiography

Transthoracic echocardiography was performed before myocardial injection and one month later using an In Vivo Microimaging System (VisualSonics, Canada) under 2.0% isoflurane inhalation. Briefly, the mice were abdominally shaved, anesthetized and placed on a heating pad. The left ventricular internal systolic dimension (LVIDs) and left ventricular internal diastolic dimension (LVIDd) were measured and averaged from three consecutive cardiac cycles. The left ventricular ejection fraction (EF) and fractional shortening (FS) were calculated. EF was calculated with the Simpson method [15], and FS was calculated as [(LVIDd-LVIDs)/LVIDs] x100%.

### Histology analysis

The mice were euthanized with 5% isoflurane, and the hearts were removed after 4 weeks. The hearts were perfused with PBS and fixed with 4% paraformaldehyde. After fixation for 24 h, the hearts were embedded in paraffin and sliced into 5 μm thick sections. Hematoxylin and eosin (H&E) staining and Picrosirius Red staining were used to evaluate cardiac morphology and collagen deposition. For immunofluorescence staining, the sections were incubated with the miR-16-2-3p probe (Servicebio, China) or with antibodies against CD31, cTnT and αSMA (all from Abcam, UK). To evaluate the microcirculation, 100 µl of FITC-lectin (Sigma, USA) at a concentration of 1 mg/ml was injected into the mice via the caudal vein to label the perfused vessels. Ten minutes after injection, heart samples were harvested to prepare frozen sections, which were subsequently immunoblotted for CD31. The histological areas of interest were all semiquantified by ImageJ software (NIH, USA).

### Cell experiments

Human aortic endothelial cells (HAECs) and human cardiac microvascular endothelial cells (HCMECs) were purchased from ATCC. The cells were cultured in Dulbecco’s modified Eagle’s medium (DMEM; Gibco, USA) supplemented with 10% fetal bovine serum (FBS; Gibco, USA) and 1% penicillin/streptomycin (P/S; Invitrogen, USA). These two cell lines were treated with high glucose (40 µM) for 48 h to mimic CAD or CMD, respectively.

### Luciferase reporter assay

A wild-type luciferase reporter was constructed with the 3’-UTR of ACADM containing miR-16-2-3p binding sites, while the mutant reporter had no binding sites. The wild-type reporter, the mutant reporter, the let-7i mimic or the negative controls were transfected into HEK293 cells using Lipo 2000 (RiboBio; China). After 24 h, the cell medium was collected for luciferase activity measurement. Firefly luciferase activity was calculated and normalized to Renilla luciferase activity.

### qRTPCR

Total RNA was extracted from cardiac tissues and cells. Subsequently, 1 µg of RNA was reverse transcribed into cDNA using HiScriptII Q RT SuperMix (Vazyme; China), and quantitative RT‒PCR was carried out with ChamQ SYBR qPCR Master Mix (Vazyme; China) following the provided protocol. All the results were normalized against glyceraldehyde-3-phosphate dehydrogenase (GAPDH) expression.

### Western blotting

Cells and exosomes were lysed with lysis buffer containing protease and phosphatase inhibitors, and the concentrations of the proteins were determined using a BCA kit (Thermo Fisher Scientific, USA). Total protein lysates were separated by SDS‒PAGE and transferred to PVDF membranes (Millipore, USA). The membranes were incubated with the corresponding primary antibodies overnight at 4 °C and then exposed to secondary antibodies for 2 h. Western blot bands were visualized using an enhanced chemiluminescence (ECL) kit (Keygene; China). The protein expression levels were normalized to the β-actin levels. The following antibodies from Abcam, USA, were utilized: Acadm, TSG101, HSP70, CD63, and CD9. The relative protein expression was quantified using ImageJ.

### Wound healing assay

HCMECs were seeded in 6-well plates to reach 90% confluence. The cells were scraped with a pipe and washed twice with PBS to remove floating cells. After 24 h, the wound was photographed again, and the scratch width was recorded. The migration distance was subsequently calculated.

### Tube formation assay

HCMECs were seeded in 12-well plates precoated with 100 µl/well of Matrigel (Corning, USA). After 8 h of incubation at 37 °C, morphological images of the tubes were captured using a computer-assisted microscope. The number of junctions and vessel length and area were analyzed using ImageJ.

### Lipidomics

Lipids were extracted according to the modified method of Bligh and Dyer. Briefly, 50 mg heart tissue samples were homogenized in 500 µl cold phosphate-buffered saline (PBS). Then, lipids were extracted by adding chloroform:methanol (2:1, v/v). Glass tubes (5 mL) were used to avoid polymer contamination. The samples were vortexed for 2 min, followed by 5 min of centrifugation at 1000 rpm after they had allowed to sit still for 20 min. The lower chloroform layer was transferred to a glass syringe and subsequently dried under nitrogen. Lipid samples were subjected to liquid chromatography‒electrospray ionization tandem mass spectrometry (Thermo, CA).

### Statistical analysis

The statistical analysis was conducted using IBM SPSS Statistics version 25.0. Normally distributed data are expressed as the mean ± SD. One-way ANOVA was used for group comparisons. GraphPad Prism 8.0 (GraphPad; United States) was used to generate figures based on the statistical analysis. A *p* value < 0.05 was considered to indicate statistical significance.

## Results

### Characterization of serum-derived exosomes from three groups of patients

The clinical characteristics of the age- and sex-matched patients are presented in Table 1. No statistically significant differences were observed among the three groups (3 patients per group) in terms of BMI, smoking status, hypertension, fasting glucose, hsCRP, renal function or lipid levels, indicating that the interference factors related to vascular complications were excluded.

**Table 1:** Clinical characteristics of patients with DM, DM-CMD and DM-CAD used for microRNA array analysis of serum derived exosomes

	DM (*n* = 3)	DM-CMD (*n* = 3)	DM-CAD (*n* = 3)	*P* value
Age	53.7 ± 13.6	56.0 ± 13.0	56.7 ± 14.1	0.96
Male	3	3	3	-
BMI	23.9 ± 2.3	24.3 ± 4.3	23.0 ± 0.9	0.86
Smoking	1	2	1	0.29
Hypertension	1	1	2	0.41
Fasting glucose	8.1 ± 2.9	6.4 ± 3.1	5.4 ± 0.5	0.47
HbA1c	9.0 ± 3.0	6.7 ± 1.1	6.8 ± 1.1	0.34
eGFR	123.7 ± 13.1	115.0 ± 21.7	118.4 ± 11.0	0.80
TC	4.7 ± 0.5	3.5 ± 1.4	2.8 ± 0.6	0.11
TG	1.12 ± 0.10	1.27 ± 0.61	1.13 ± 0.21	0.86
hsCRP	2.98 ± 0.31	1.85 ± 1.18	1.40 ± 0.43	0.10
Metformin	3	0	2	-
Sglt2i	0	1	2	-

BMI: body mass index; HbA1c: Hemoglobin A1C; eGFR: estimated glomerular filtration rate; TC: total cholesterol; TG: triglyceride; hsCRP: high-sensitivity C-reactive protein; Sglt2i: Sodium-Glucose Transport Protein 2 inhibitor

**Fig. 1:**
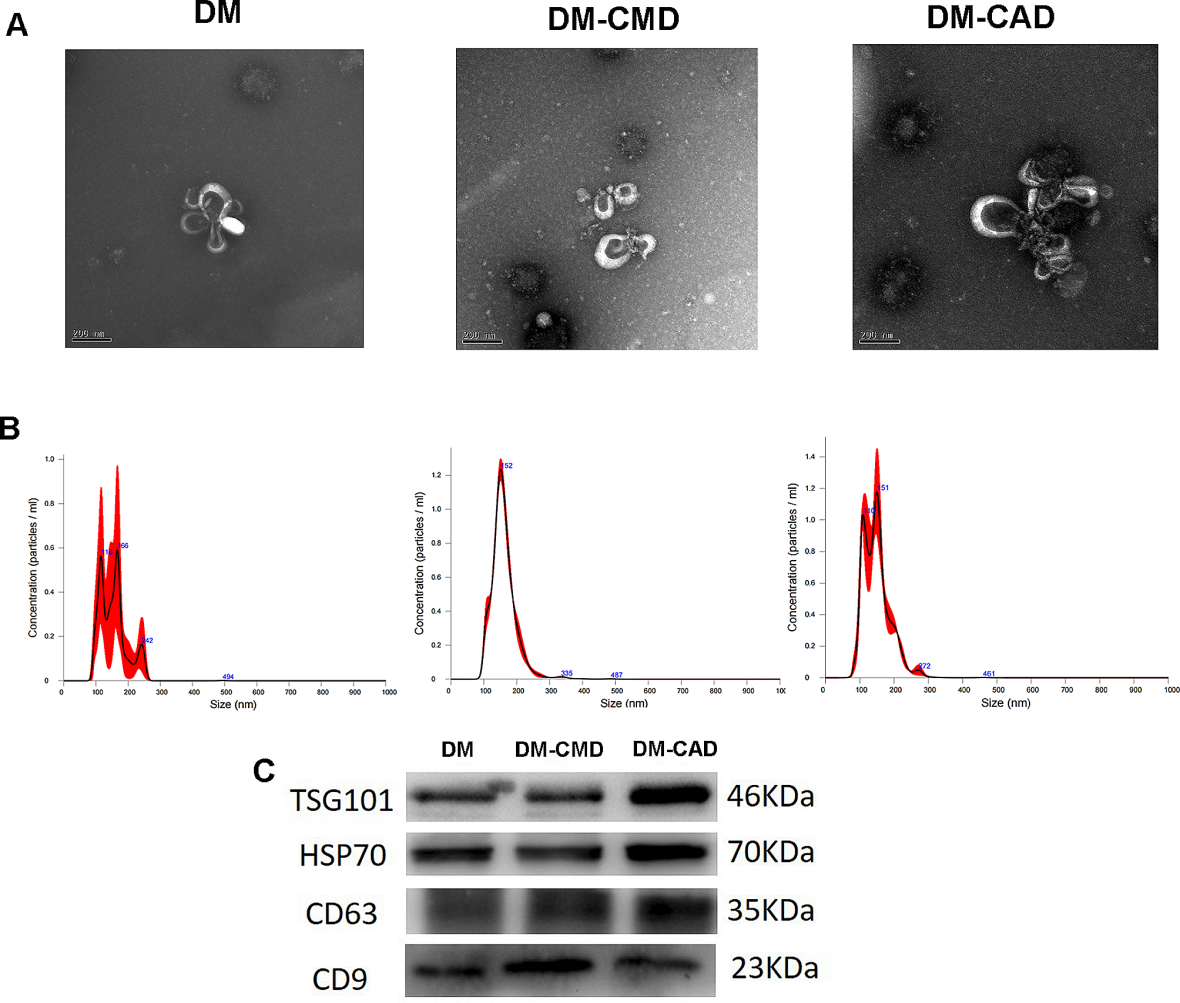
Isolation and characterization of serum-derived exosomes from DM, DM-CMD and DM-CAD. (**a**) Representative transmission electron microscopy (TEM) images of serum-derived exosomes isolated using the ExoQuick method (scale bar, 100 nm). (**b**) Representative images of the nanoparticle tracking analysis (NTA) of serum-derived exosomes, indicating the concentration and size distribution of the isolated particles. (**c**) Representative immunoblot images of the exosomal markers TSG101, HSP70, CD63 and CD9 (*n* = 3/group)

Exosomes were extracted from the serum of the three groups. Transmission electron microscopy (TEM) confirmed that the cells had circular or cup-shaped morphologies with a diameter of approximately 150 nm (Fig. 1A). Nanoparticle tracking analysis (NTA) revealed that exosomes from the DM group were present at greater concentrations than were those from the other two groups (Table 2). The sizes of the particles ranged from 110 to 160 nm (Fig. 1B). Western blotting indicated that the exosomes expressed the surface markers TSG101, HSP70, CD63 and CD9 (Fig. 1C). The characterization of morphology, size and surface marker profiles supported successful isolation of exosomes from serum.

**Table 2:** Particle concentration, size and protein concentration of serum-derived exosomes

Groups	Particle concentration (x10^9/ml)	Mean size (nm)	Protein concentration (mg/ml)
DM	1.25	154.1	0.91
DM-CMD	0.83	160.6	1.14
DM-CAD	0.91	149.4	1.10

### Mir-16-2-3p was upregulated in serum-derived exosomes from DM-CMD

**Fig. 2:**
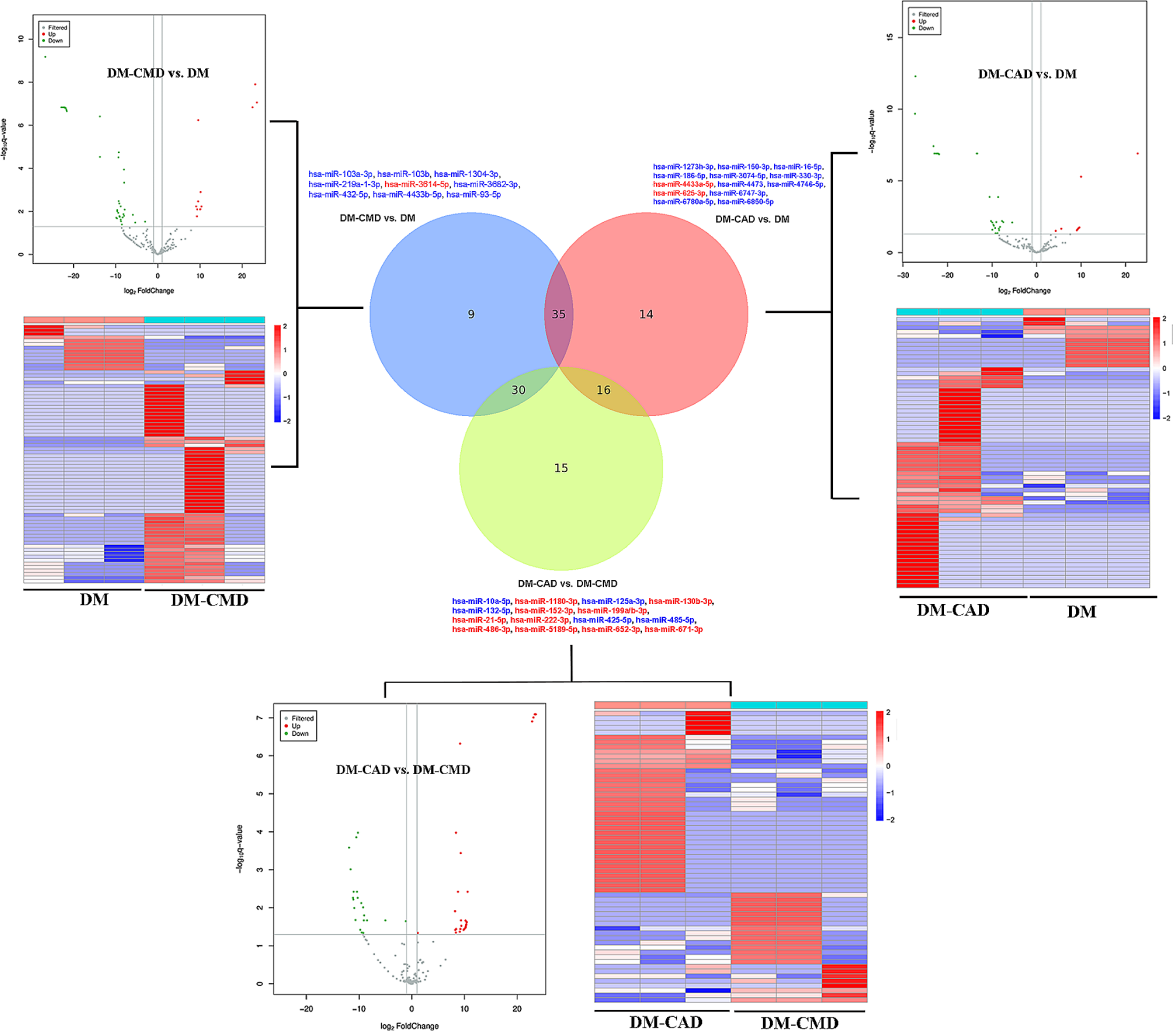
Venn diagram showing unique and common miRNAs in different groups and differentially expressed miRNAs. The Venn diagram shown here represents miRNAs with average read counts > 100 reads/million used for comparisons among DM, DM-CMD and DM-CAD. Pairwise comparisons showing differences in the expression of miRNAs among DM, DM-CMD and DM-CAD. The differentially expressed miRNAs across two different comparisons are indicated by red (upregulated) and green (downregulated) coloring. Volcano plots showing the differentially expressed miRNAs identified via pairwise comparisons. Red indicates genes with a fold change > 2 and a q value < 0.05, while blue indicates genes with a fold change < 0.5 and a q value < 0.05. Heatmap clustering of the differentially expressed miRNAs significant among groups (*n* = 3/group)

Given that the morphology, size, and marker profile were comparable between the groups (3 patients per group), our focus was shifted to exploring the enriched miRNA profiles by high-throughput sequencing. Table 3 displays the ten most abundant miRNAs detected in the serum-derived exosomes among the groups. The pairwise comparisons are presented in Fig. 2.

**Table 3:** Ten most abundant miRNAs detected in serum-derived exosomes among groups

DM			DM-CMD			DM-CAD		
Rank	miRNAs	Average	Rank	miRNAs	Average	Rank	miRNAs	Average
1	hsa-let-7b-5p	13892.72	1	hsa-let-7b-5p	12190.69	1	hsa-let-7b-5p	13206.47
2	hsa-miR-151a-3p	8926.46	2	hsa-miR-151a-3p	7696.053	2	hsa-miR-486-5p	8661.416
3	hsa-let-7a-5p	6584.443	3	hsa-miR-486-5p	6684.649	3	hsa-miR-151a-3p	7963.141
4	hsa-miR-486-5p	6175.946	4	hsa-miR-423-5p	3654.069	4	hsa-miR-423-5p	4528.686
5	hsa-miR-423-5p	3260.354	5	hsa-miR-10b-5p	1959.278	5	hsa-let-7a-5p	2199.651
6	hsa-let-7f-5p	1876.894	6	hsa-let-7a-5p	1545.363	6	hsa-miR-122-5p	1886.831
7	hsa-miR-148a-3p	1819.63	7	hsa-miR-148a-3p	1279.185	7	hsa-miR-10b-5p	1620.325
8	hsa-miR-451a	991.2441	8	hsa-miR-10a-5p	1098.301	8	hsa-miR-148a-3p	1298.461
9	hsa-miR-10b-5p	986.6856	9	hsa-miR-122-5p	736.9085	9	hsa-miR-92a-3p	760.4896
10	hsa-miR-10a-5p	507.1821	10	hsa-miR-92a-3p	630.8218	10	hsa-miR-10a-5p	580.172

**Fig. 3:**
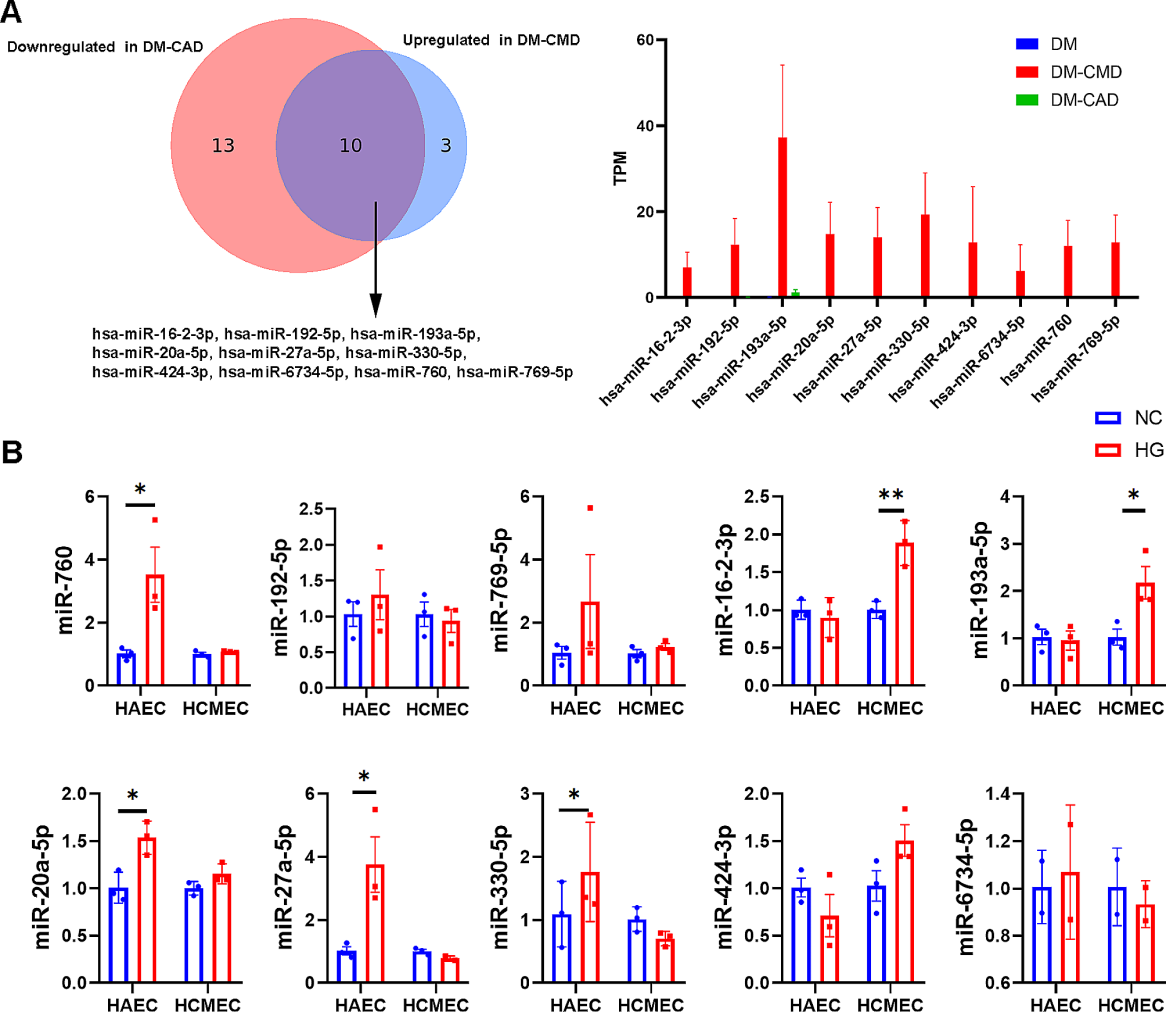
(**a**) We selected 10 candidate miRNAs specific to DM-CMD that were downregulated in DM-CAD but upregulated in DM-CMD. (**b**) We detected the expression of these 10 miRNAs in high glucose-treated coronary artery endothelial cells (HAECs) and cardiac microvascular endothelial cells (HCMECs). *P < 0.05, **P < 0.01, ***P < 0.001; *n* = 3 replicates/group

To identify specific miRNAs associated with diabetic coronary microvascular dysfunction, we screened miRNAs downregulated in DM-CAD patients (vs. DM) and upregulated in DM-CMD patients (vs. DM). The following ten miRNAs met the inclusion criteria: hsa-miR-16-2-3p, hsa-miR-192-5p, hsa-miR-193a-5p, hsa-miR-20a-5p, hsa-miR-27a-5p, hsa-miR-330-5p, hsa-miR-424-3p, hsa-miR-6734-5p, hsa-miR-760 and hsa-miR-769-5p (Fig. 3A). To further validate the specific miRNAs associated with diabetic coronary microvascular dysfunction, we conducted experiments using high glucose-treated human aortic endothelial cells (HAECs) and human cardiac microvascular endothelial cells (HCMECs) to simulate CAD and CMD, respectively (3 replicates per group). Q-PCR revealed that only miR-16-2-3p and miR-193a-5p exhibited significantly greater expression in HMECs, indicating their potential role in diabetic coronary microvascular dysfunction (Fig. 3B). Given that miR-193a-5p has been reported to be associated with diabetic nephropathy [16], we selected miR-16-2-3p for further exploration.

### Mir-16-2-3p was downregulated in cardiac tissues

**Fig. 4:**
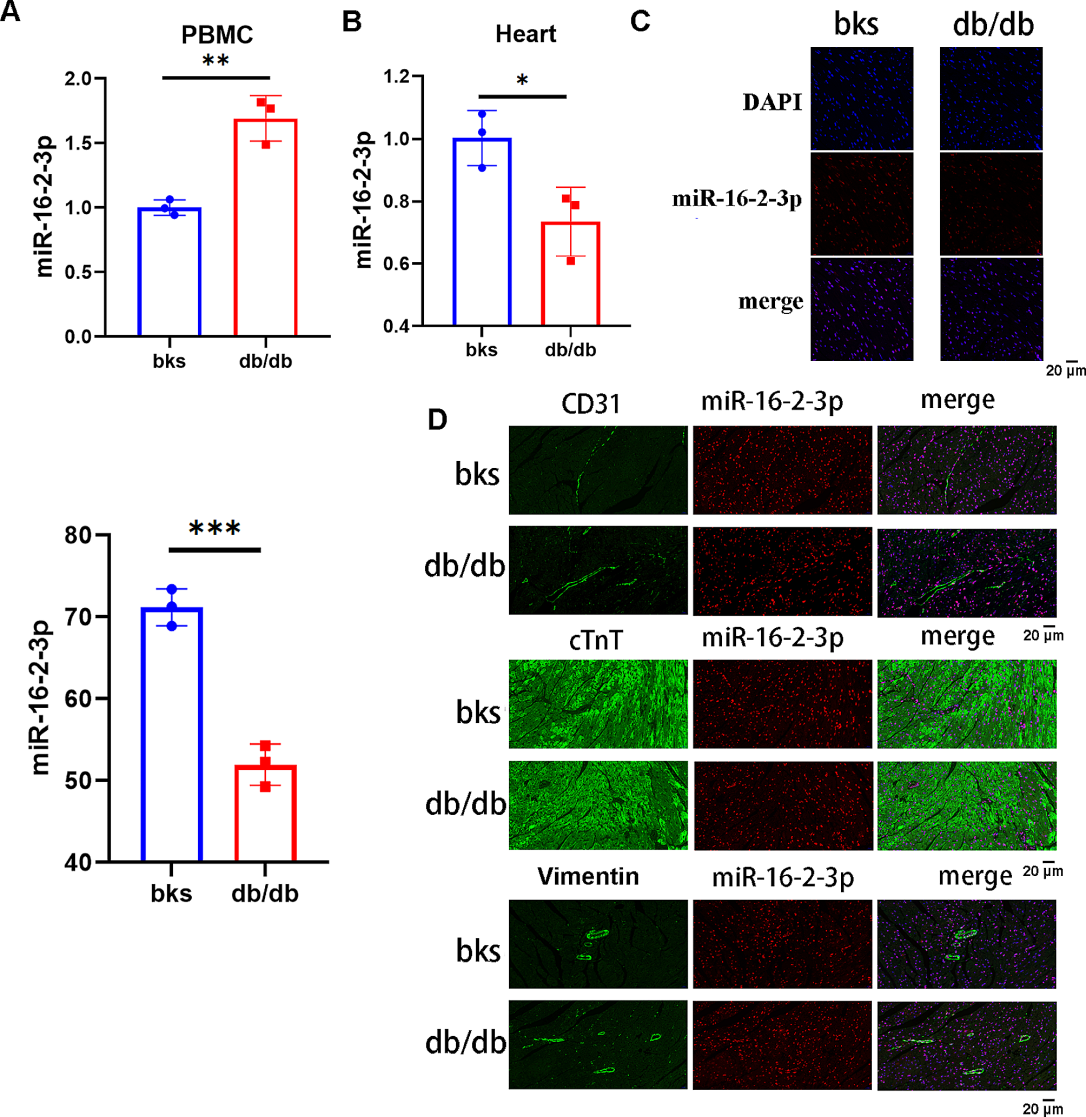
The expression of miR-16-2-3p in (**a**) peripheral blood mononuclear cells and (**b**) cardiac tissue. (**c**) FISH further confirmed the expression of miR-16-2-3p in cardiac tissues. (**d**) Immunofluorescence staining of the miR-16-2-3p probe for CD31, cTnT, and vimentin. *P < 0.05, **P < 0.01, ***P < 0.001; *n* = 3 mice/group

Q-PCR revealed significantly greater expression of miR-16-2-3p in the PBMCs of db/db mice (3 mice per group), while a contrasting trend was observed in the myocardium of db/db mice (Fig. 4A**&B**). Fluorescence in situ hybridization (FISH) further confirmed that miR-16-2-3p was expressed at lower levels in the myocardium of db/db mice (Fig. 4C). Next, we aimed to explore the cardiac cell-specific localization of miR-16-2-3p in CD31^+^ endothelial cells, cTnT^+^ cardiomyocytes and vimentin^+^ fibroblasts. The results indicated that the expression of miR-16-2-3p was comparable among the main cell types (Fig. 4D). Therefore, we inferred that the serum exosomal miR-16-2-3p may be derived from cardiac tissues, although the specific cardiac cell types should be determined by single-cell sequencing.

### Cardiac-specific overexpression of mir-16-2-3p improved microvascular dysfunction

**Fig. 5:**
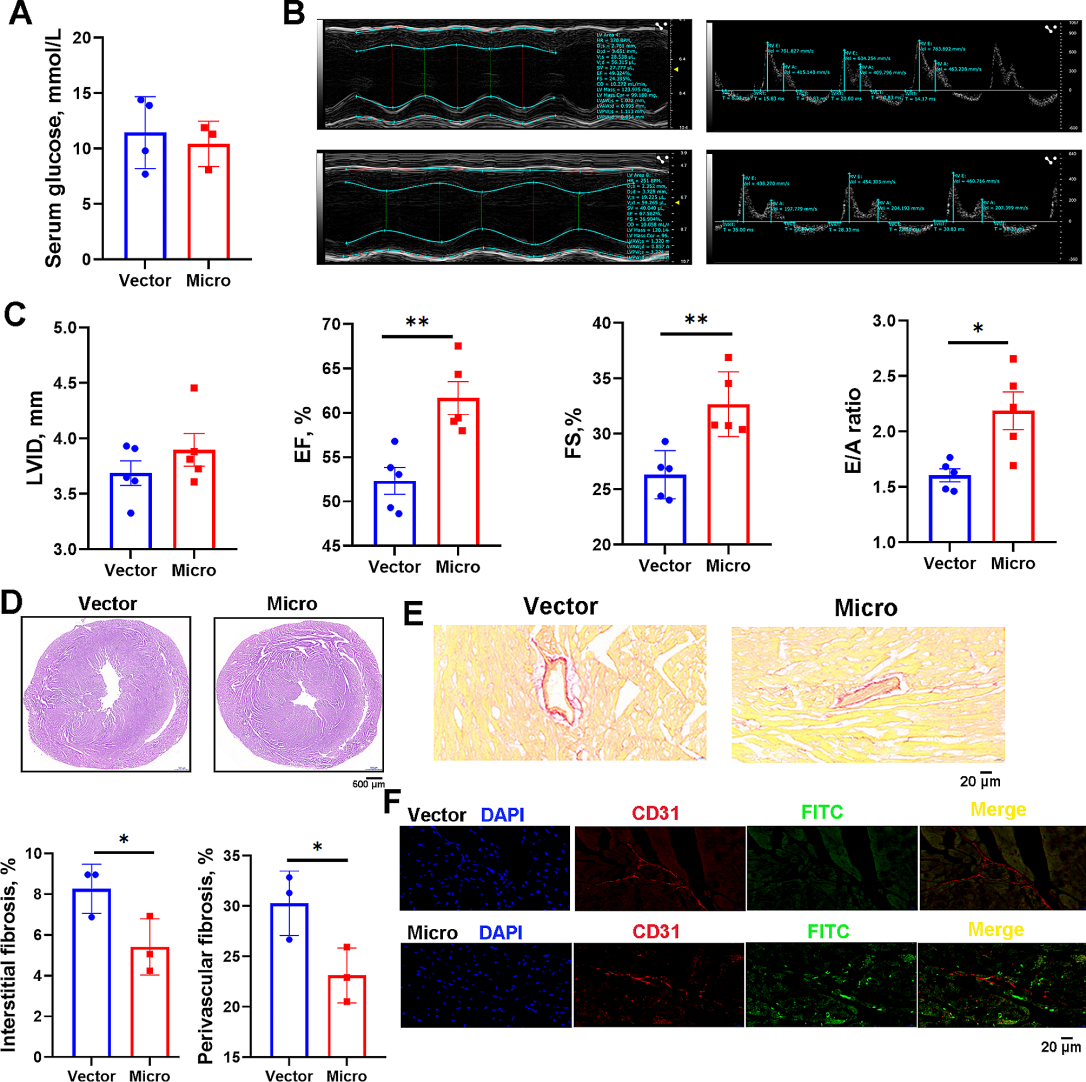
db/db mice received an intramyocardial injection of lentivirus overexpressing miR-16-2-3p. One month later, the glucose level (**a**), cardiac function (**b, c**), pathological morphology (**d**), interstitial fibrosis and perivascular fibrosis (**e**) were evaluated. Mice were injected with FITC-labeled lectin to evaluate the microcirculation (**f**). *P < 0.05, **P < 0.01, ***P < 0.001; *n* = 5 mice/group

Db/db mice received an intramyocardial injection of lentivirus to overexpress miR-16-2-3p (microgroup; 5 mice per group). The serum glucose levels were comparable at approximately 10 mmol/L after one month (Fig. 5A). Echocardiography was performed to evaluate cardiac function (Fig. 5B). EF%, FS% and the E/A ratio were significantly greater in the Micro group than in the control group, indicating that cardiac contractile and diastolic function were ameliorated (Fig. 5C). The morphology of the hearts was comparable between the two groups, as shown by H&E staining (Fig. 5D). Sirius red staining showed a noticeable alleviation of interstitial and perivascular fibrosis in the Micro group (Fig. 5E). Moreover, mice were intravenously injected with FITC-labeled lectin to evaluate cardiac microcirculation. A brighter green fluorescence was observed in the Micro group (Fig. 5F). Taken together, the overexpression of miR-16-2-3p in db/db mouse hearts was associated with improved cardiac function, fibrosis and microcirculation.

### Mir-16-2-3p was involved in fatty acid degradation by targeting ACADM

**Fig. 6:**
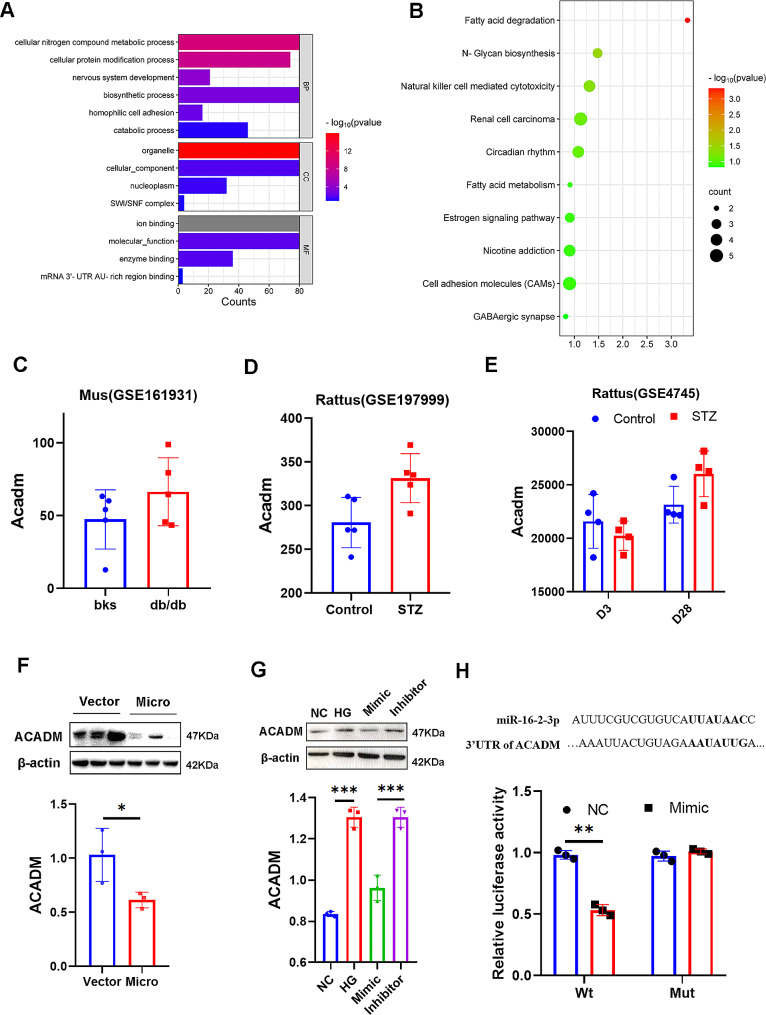
GO (**a**) and KEGG (**b**) enrichment analyses of miR-16-2-3p. (**c, d, e**) The expression of ACADM in diabetic patients according to public databases. The protein expression of ACADM in miR-16-2-3p-overexpressing cardiac tissues (**f**) and microvascular endothelial cells (**g**). The luciferase reporter assay (**h**). *P < 0.05, **P < 0.01, ***P < 0.001; *n* = 3 replicates/group

Functional enrichment analysis was performed to elucidate the underlying biological functions of miR-16-2-3p. The results indicated its potential involvement in fatty acid degradation, with ACADM being one of its target genes (Fig. 6A**&B**). Correspondingly, we observed upregulation of ACADM in both type 1 and type 2 diabetes patients by analyzing public databases (Fig. 6C, D**&E**). Western blotting also demonstrated that overexpression of miR-16-2-3p reduced the expression of cardiac ACADM (Fig. 6F). In vitro assays also revealed that overexpressing miR-16-2-3p reduced the expression of ACADM in microvascular endothelial cells (3 replicates per group; Fig. 6G). Furthermore, the luciferase assay confirmed the direct interaction of miR-16-2-3p with ACADM, and the binding sequence was nucleic acid UUAUAAC (Fig. 6H).

**Fig. 7:**
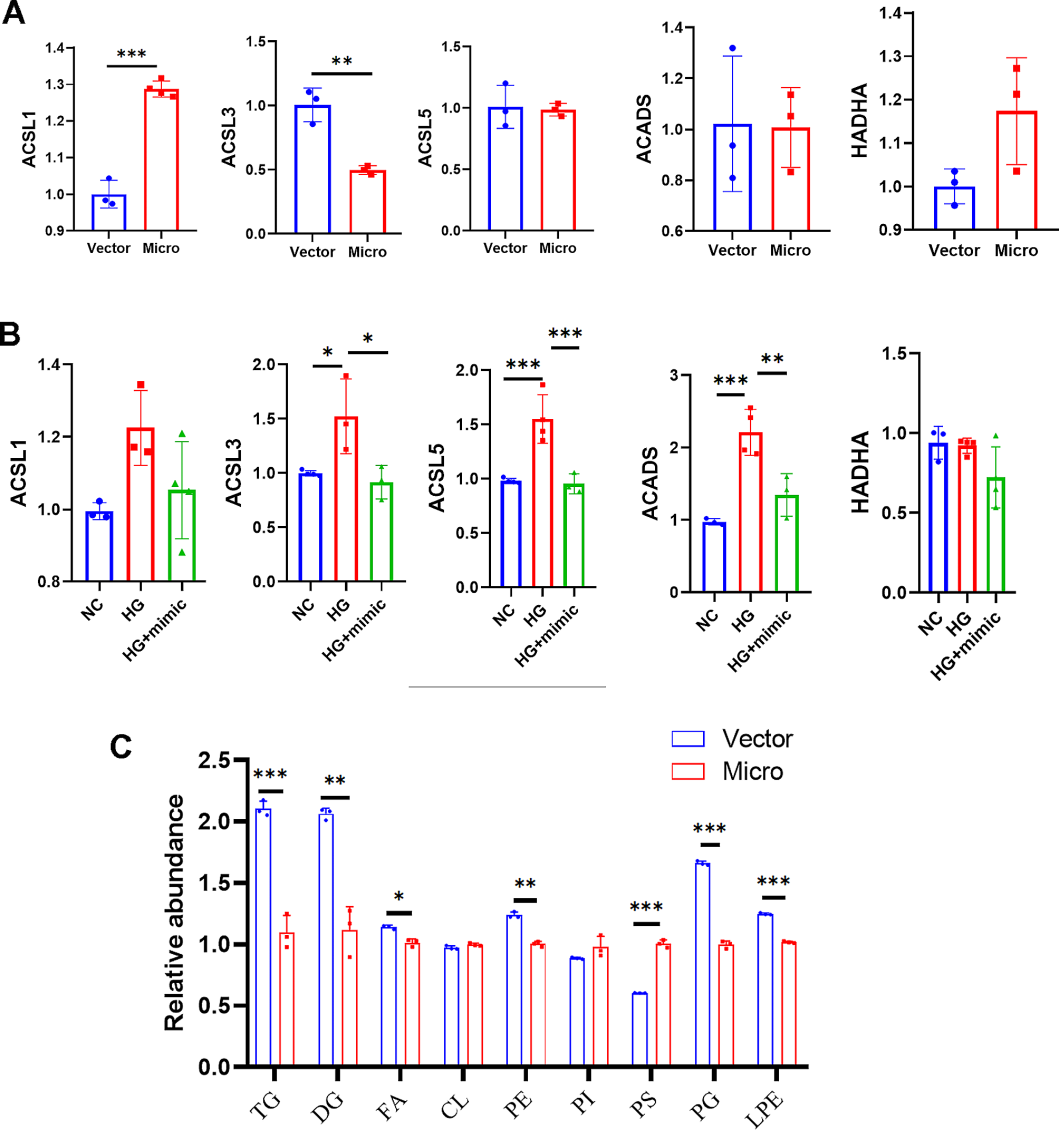
The expression of genes related to fatty acid degradation pathways both in vivo (**a**) and in vitro (**b**). Lipidomic analysis of the two groups (**c**). *P < 0.05, **P < 0.01, ***P < 0.001; *n* = 3 replicates/group

Furthermore, we detected the expression of genes related to the fatty acid degradation pathway in vivo and in vitro (3 replicates per group) and found that overexpression of miR-16-2-3p could regulate the fatty acid degradation pathway (Fig. 7A**&B**). Lipidomic analysis also revealed that the level of medium-long fatty acids was decreased in the miR-162-3p-overexpressing group (Fig. 7C).

### miR-16-2-3p alleviated inflammation but enhanced tube formation in microvascular endothelial cells

**Fig. 8:**
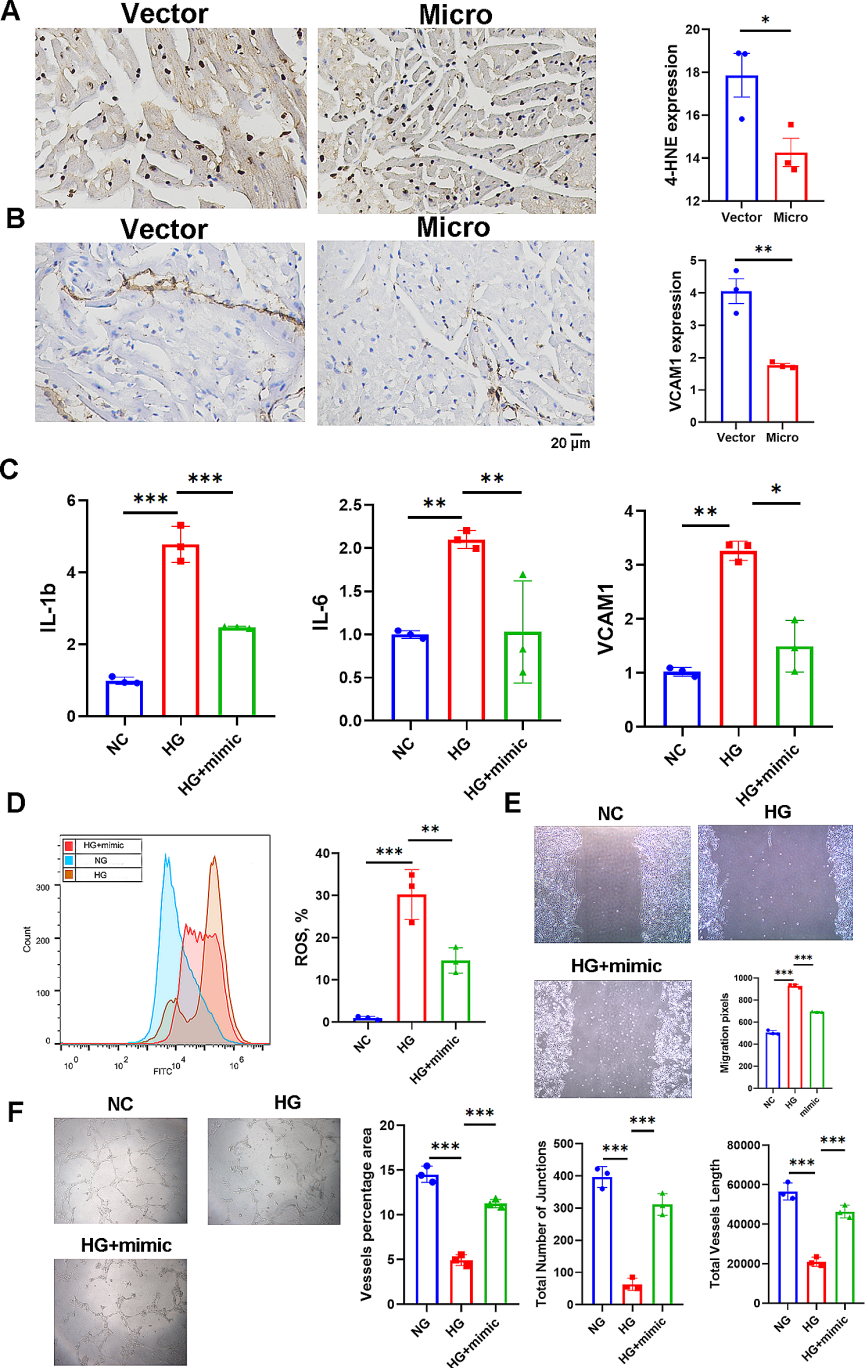
Immunohistochemistry was used to evaluate the expression of 4-HNE (**a**) and VCAM1 (**b**) in cardiac tissues. The mRNA expression of inflammatory cytokines (**c**). Reactive oxidative stress was assessed by flow cytometry (**d**). Wound healing assay (**e**) and tube formation assay (**f**). *P < 0.05, **P < 0.01, ***P < 0.001; *n* = 3 replicates/group

Immunohistochemical staining indicated that overexpression of miR-16-2-3p reduced the expression of 4-HNE and VCAM1, suggesting that miR-16-2-3p alleviated oxidative stress and the inflammatory response (Fig. 8A**&B**). In vitro assays also suggested that miR-16-2-3p inhibited the expression of inflammatory cytokines, such as IL-1β, IL-6 and VCAM1 (Fig. 8C), and oxidative stress (Fig. 8D). To explore the impact of miR-16-2-3p on the function of microvascular endothelial cells, we investigated cell migration and tube formation ability. According to the wound healing assay, the migration distance of the HCMECs was increased in the overexpression group (Fig. 8E). Representative images of tubes formed by HCMECs in each group are shown in Fig. 8F. These findings suggested that miR-16-2-3p increased the tube-forming capacity, as evidenced by the vessel percentage area, total number of junctions and total vessel length.

## Discussion

In this study, we isolated exosomes and profile-enriched miRNAs from the serum of DM, DM-CAD and DM-CMD patients. We found that miR-16-2-3p was upregulated in microvascular endothelial cells from DM-CMD- and high glucose-treated patients. Moreover, cardiac miR-16-2-3p overexpression improved cardiac diastolic function and coronary microvascular dysfunction in db/db mice. Finally, we discovered that miR-16-2-3p may regulate ACADM-mediated fatty acid degradation in endothelial cells.

Indeed, CMD not only is a simple disease but is also is involved in the development of other cardiovascular diseases [17]. Recently, many studies have confirmed the relationship between CMD and heart failure with preserved ejection fraction [18, 19]. Christopher J. Rush et al. reported that 81% of patients with HFpEF but without CAD had CMD [20]. Another study demonstrated that 75% of patients with HFpEF had CMD [18]. These studies suggested a high prevalence of CMD in HFpEF patients. Patients with HFpEF and endothelium-independent CMD reportedly exhibit worsened diastolic dysfunction and outcomes [21]. In this study, we found significant improvement in cardiac diastolic function in db/db mice through cardiac overexpression of miR-16-2-3p. CMD was also ameliorated, as evaluated with FITC-labeled lectin. These findings suggested that miR-16-2-3p could be a therapeutic target for CMD.

Fatty acid metabolism is a complex process that includes fatty acid uptake and oxidation [22]. Abnormal fatty acid metabolism has been implicated in diabetes, as evidenced by increased levels of fatty acids and elevated myocardial fatty acid oxidation [23, 24]. An increase in fatty acid oxidation could result in the accumulation of reactive oxygen species, ultimately decreasing the capacity of the myocardium to oxidize fatty acids, while this reduced oxidative capacity could result in lipid accumulation, triggering the detrimental effects that have been associated with lipotoxicity [25, 26]. While previous studies have focused on fatty acid metabolism in cardiomyocytes [27], our study confirmed that circulating exosomal miR-16-2-3p/ACADM may regulate fatty acid degradation in microvascular endothelial cells.

Vascular endothelial cells form the inner layer of blood vessels and maintain the balance of the circulatory system [28]. The accumulation of ROS [29, 30] and inflammatory signals [31] and disorders of cellular metabolic pathways in endothelial cells contribute to microvascular dysfunction. Endothelial cells primarily acquire ATP via the glycolytic pathway and, to a much lesser extent, from fatty acid metabolism [32]. In some situations, such as high glucose levels [33], insulin resistance [34] and cancer [35], fatty acid degradation increases in endothelial cells [36]. It is believed that fatty acid metabolism regulates redox balance by producing NADPH [37]. In addition, fatty acid metabolism controls endothelial cell function [38] and blood vessel stability [39]. We found that miR-16-2-3p participated in fatty acid degradation and targeted ACADM through enrichment analysis. ACADM, an acyl-CoA dehydrogenase medium chain gene, is a medium-chain fatty acid oxidant and is upregulated in both type 1 and type 2 diabetes patients, as analyzed from public data. In both in vivo and in vitro studies, we found that the overexpression of miR-16-2-3p led to the downregulation of ACADM. Furthermore, overexpression of miR-16-2-3p could alleviate oxidative stress and inflammation in vascular cells. Consistent with our findings, Shawn et al. also reported that inhibiting elevated fatty acid β-oxidation in endothelial cells improved cardiac diastolic function in CMD [40].

The methodologies employed in the isolation and characterization of exosomes are integral to the validity of our findings. However, it is crucial to acknowledge the existing controversies and challenges associated with these techniques. The potential for contamination, coisolation of other extracellular vesicles, and impact on exosome integrity are noteworthy concerns. Moreover, the lack of standardized protocols for characterization methods contributes to variability due to the heterogeneity of exosomes in terms of size, surface markers, and cargo content.

## Conclusion

In this study, we found that miR-16-2-3p was upregulated in the circulating exosomes of DM-CMD patients. Furthermore, miR-16-2-3p could alleviate coronary microvascular dysfunction in diabetes through regulating the fatty acid degradation of endothelial cells. Our results suggested that circulating miR-16-2-3p could be a potential biomarker and therapeutic target in DM-CMD.

### Electronic supplementary material

Below is the link to the electronic supplementary material.

Supplementary Material 1

## Data Availability

No datasets were generated or analysed during the current study.
