# Supplementary figures and images for: Circulating exosomal mir-16-2-3p is associated with coronary microvascular dysfunction in diabetes through regulating the fatty acid degradation of endothelial cells
Source: Cardiovasc Diabetol. 2024 Feb 9;23:60. doi: 10.1186/s12933-024-02142-0 (PMC10858495; doi:10.1186/s12933-024-02142-0)

## Slide 1
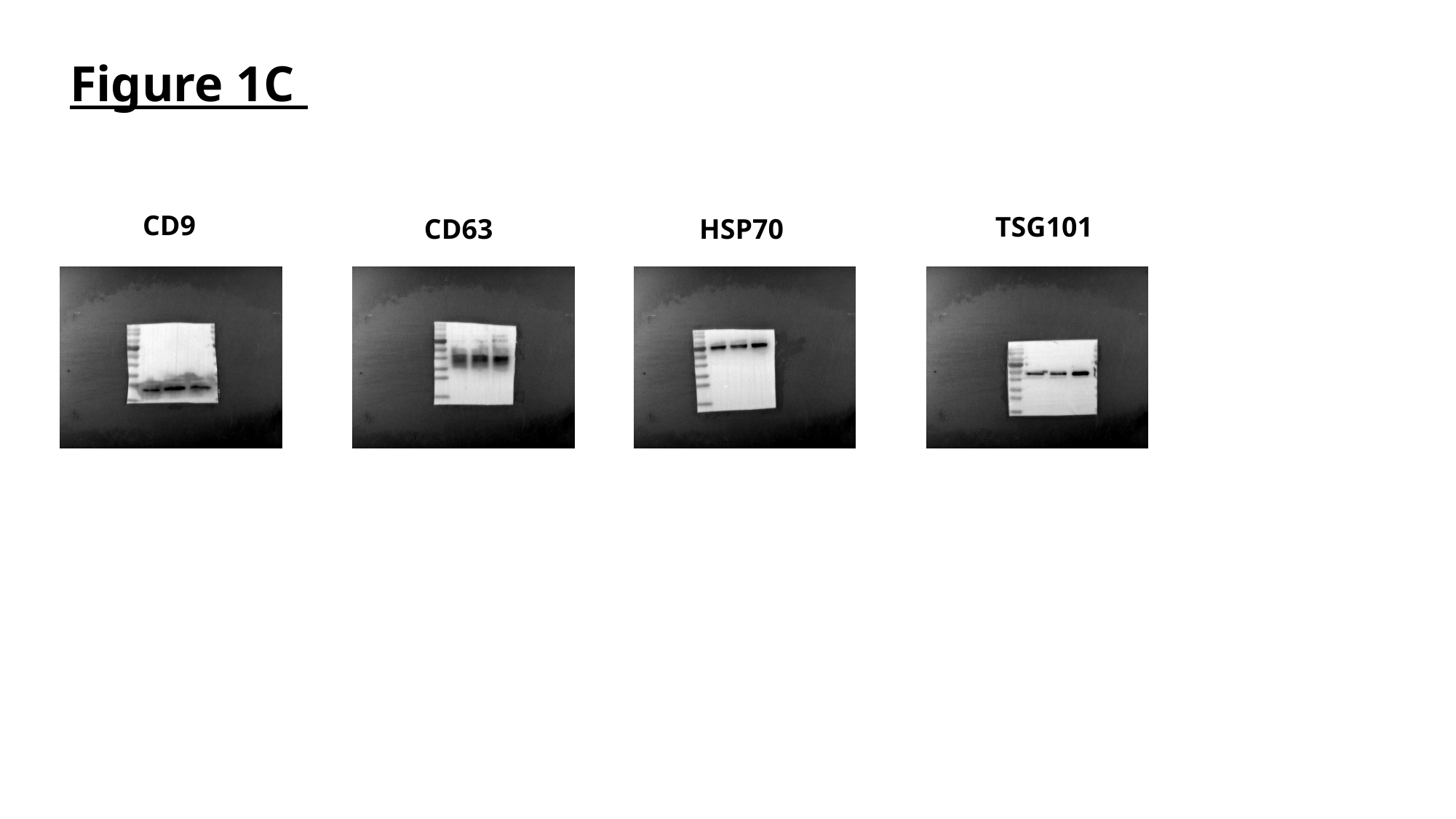

Figure 1C
CD9
TSG101
CD63
HSP70

## Slide 2
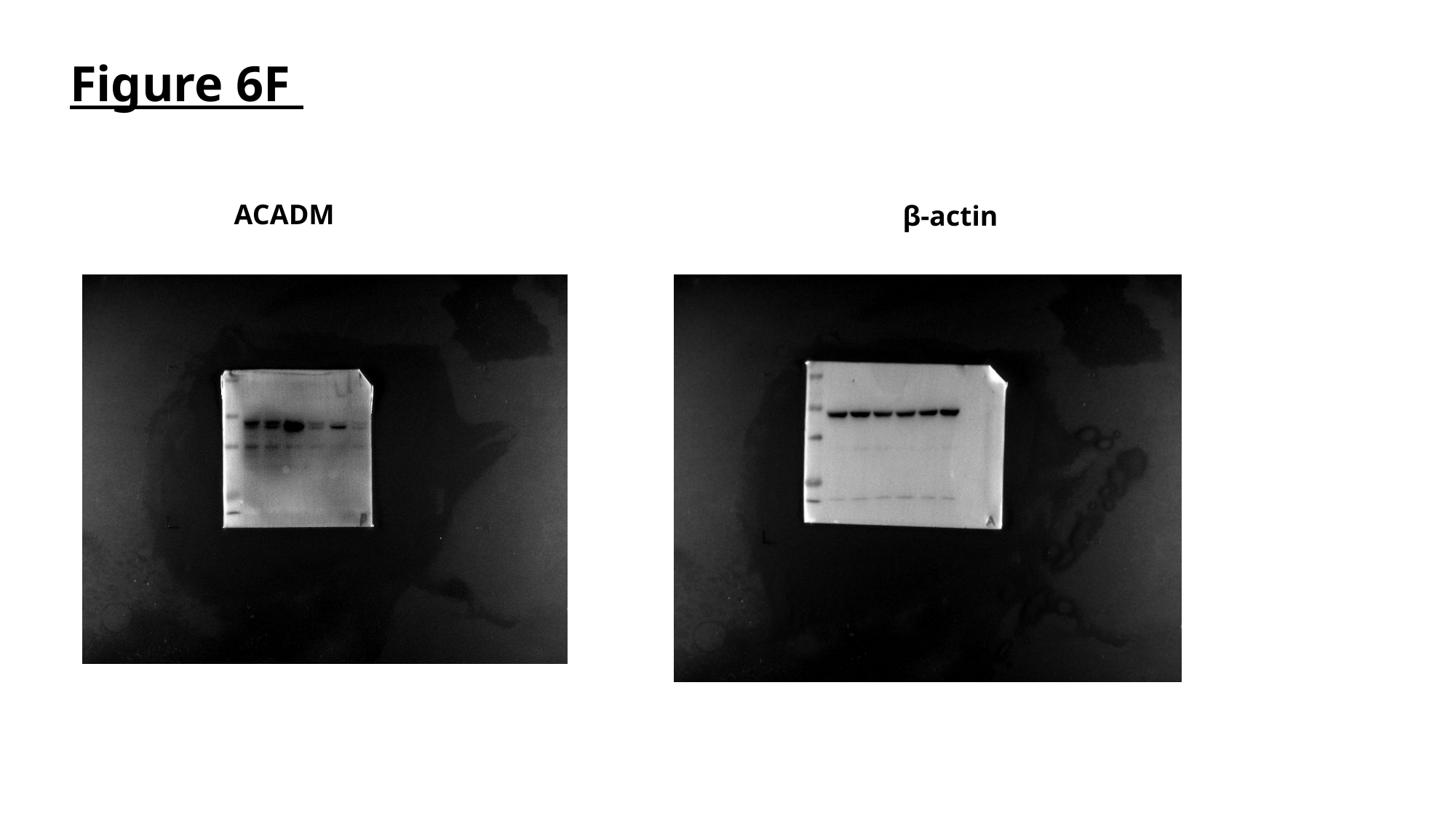

Figure 6F
ACADM
β-actin

## Slide 3
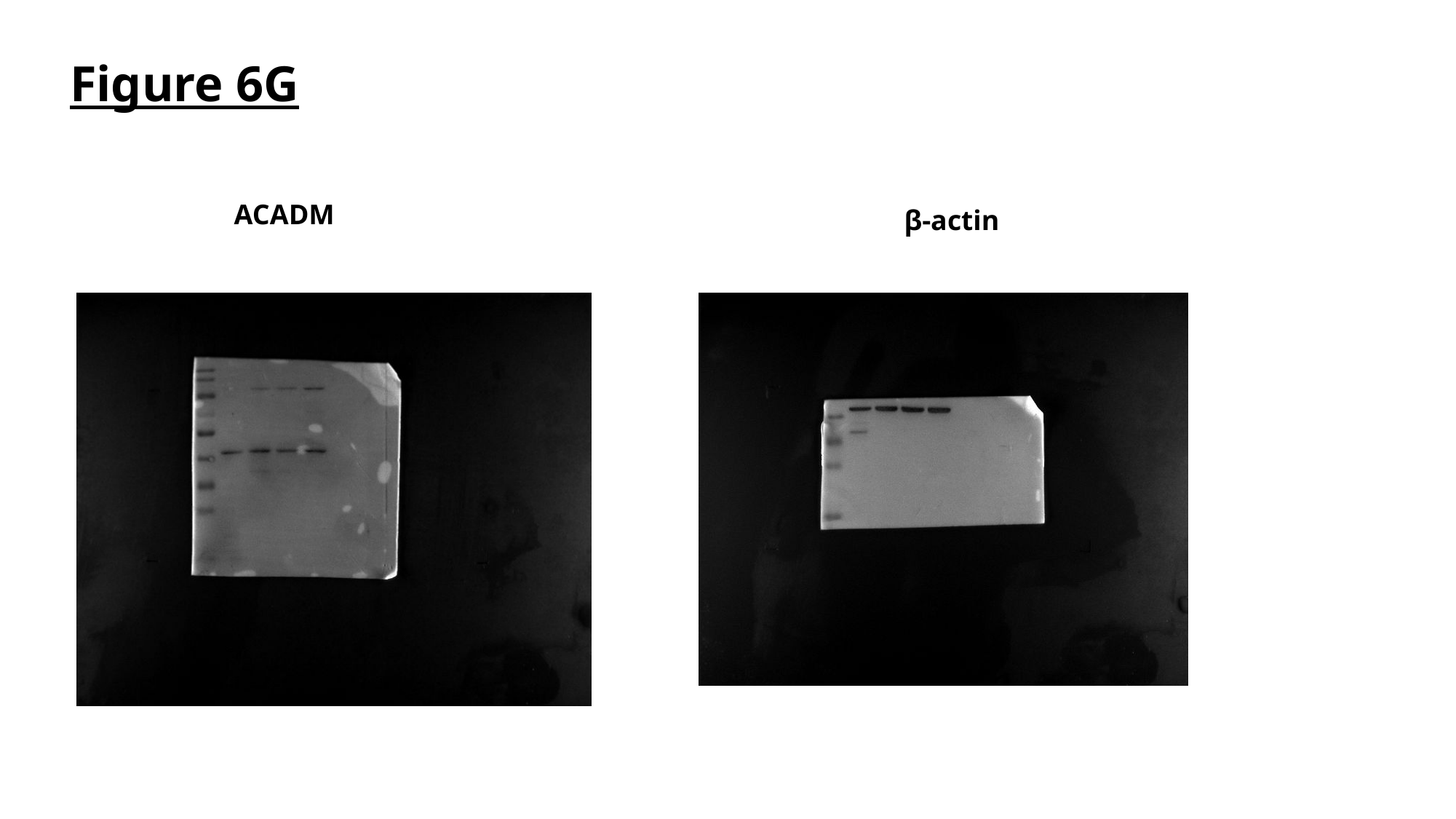

Figure 6G
ACADM
β-actin

Supplement: Supplementary file 1 — Supplementary Material 1 [file 12933_2024_2142_MOESM1_ESM.pptx]
